# Psychoactive pharmaceuticals at environmental concentrations induce in vitro gene expression associated with neurological disorders

**DOI:** 10.1186/s12864-016-2784-1

**Published:** 2016-06-29

**Authors:** Gaurav Kaushik, Yu Xia, Luobin Yang, Michael A. Thomas

**Affiliations:** Department of Biological Sciences, Idaho State University, Stop 8007, 921S 8th Ave, Pocatello, ID 83209-8007 USA; Department of Medical Pathology and Laboratory Medicine, University of California at Davis, Davis, CA 95817 USA; Present Address: Institute for Pediatric Regenerative Medicine, Shriners Hospitals for Children, Northern California, 2425 Stockton Boulevard, Sacramento, CA 95817 USA; Division of Biological Sciences, University of Montana, 32 Campus Dr, Missoula, MT 59812 USA

## Abstract

**Background:**

A number of researchers have speculated that neurological disorders are mostly due to the interaction of common susceptibility genes with environmental, epigenetic and stochastic factors. Genetic factors such as mutations, insertions, deletions and copy number variations (CNVs) are responsible for only a small subset of cases, suggesting unknown environmental contaminants play a role in triggering neurological disorders like idiopathic autism. Psychoactive pharmaceuticals have been considered as potential environmental contaminants as they are detected in the drinking water at very low concentrations. Preliminary studies in our laboratory identified gene sets associated with neuronal systems and human neurological disorders that were significantly enriched after treating fish brains with psychoactive pharmaceuticals at environmental concentrations. These gene expression inductions were associated with changes in fish behavior. Here, we tested the hypothesis that similar treatments would alter in vitro gene expression associated with neurological disorders (including autism) in human neuronal cells. We differentiated and treated human SK-N-SH neuroblastoma cells with a mixture (fluoxetine, carbamazepine and venlafaxine) and valproate (used as a positive control to induce autism-associated profiles), followed by transcriptome analysis with RNA-Seq approach.

**Results:**

We found that psychoactive pharmaceuticals and valproate significantly altered neuronal gene sets associated with human neurological disorders (including autism-associated sets). Moreover, we observed that altered expression profiles in human cells were similar to gene expression profiles previously identified in fish brains.

**Conclusions:**

Psychoactive pharmaceuticals at environmental concentrations altered in vitro gene expression profiles of neuronal growth, development and regulation. These expression patterns were associated with potential neurological disorders including autism, suggested psychoactive pharmaceuticals at environmental concentrations might mimic, aggravate, or induce neurological disorders.

**Electronic supplementary material:**

The online version of this article (doi:10.1186/s12864-016-2784-1) contains supplementary material, which is available to authorized users.

## Background

There are approximately 3000 synthetic chemicals known to interact with humans through air, water, and food [1, 2]. These chemicals might serve as environmental contaminants and trigger neurological disorders, such as autism spectrum disorders (ASD), in genetically susceptible individuals [3–5]. Among this diverse group of environmental contaminants, we focused on pharmaceuticals and personal care products (PPCPs) due to their tendency to contaminate environmental water systems [2]. PPCPs include psychoactive pharmaceuticals that are highly prescribed in the United States and other chemicals like bis-phenol A (BPA) in plastics, phthalates in cosmetics and teratogenic chemicals [1].

Psychoactive pharmaceuticals like fluoxetine, venlafaxine and carbamazepine, have been detected in the drinking water at very low concentrations [2, 6, 7]. These pharmaceuticals, some of which are metabolically active and have relatively long half-lives for over a month, reach waste-water treatment plants (WWTP) through excretion by clinical patients [7, 8]. Due to the chemical properties of these drugs and inefficient filtration of WWTP, these drugs end up mixing up with the ground water, and thus reach drinking water at low concentrations [7, 8].

We previously hypothesized that psychoactive pharmaceuticals as environmental contaminants alter neuronal gene expression associated with neurological disorders like ASD. To determine this, our lab treated juvenile fathead minnows (*Pimephales promelas*) with psychoactive pharmaceuticals (fluoxetine, venlafaxine and carbamazepine) individually and in mixtures at environmentally relevant concentrations [2, 6]. After treating them for 15 days, we extracted the brains and carried out microarray analysis. Using gene set enrichment analysis (GSEA) [9], we identified enrichment (up- or down-regulation) of gene sets associated with neuronal growth, regulation and development in the juvenile minnow brains in response to psychoactive drug exposure [2, 6]. We also identified altered neuronal gene sets associated with neurological disorders, including ASD [6]. Moreover, fish exposed to psychoactive pharmaceuticals exposed had an altered behavioral phenotype [2, 10].

In the present study, we hypothesized that psychoactive pharmaceuticals (fluoxetine, venlafaxine and carbamazepine) at environmental concentrations would alter in vitro human neuronal gene expression that is 1) similar to gene expression profile in fathead minnow, and 2) associated with neuro-developmental disorders. To determine if altered gene expression profile was associated with idiopathic autism, we also treated human neuronal cells with valproic acid, which is known to induce autism-like phenotypes in mice [11]. Identifying altered gene expression profiles in treated cells and finding a similar pattern with valproic acid induced gene expression would reveal the extent to which psychoactive pharmaceuticals at very low concentrations could induce gene expression associated with potential neurological disorders like ASD.

## Methods

### Cell culture and differentiation

Human SK-N-SH cell line was obtained from American Type Culture Collection (ATCC #HTB-11). Cells were cultured in Eagle’s Minimum Essential Medium (EMEM; ATCC). This media was supplemented with 1 % penicillin-streptomycin-neomycin (Sigma) and 10 % (v/v) fetal bovine serum (FBS). Retinoic acid (RA; Sigma) was used to induce SK-N-SH cells [12] to differentiate into more neuron-like cells [13] because this cell line is a mixture of different cell types. Approximately 15,000 cells were cultured in T-75 flask (Corning) supplemented with EMEM media for two days, and then Retinoic acid (10 μM) was added. Cells were treated with RA for two weeks and media was replaced every three-four days [12]. Cultures were monitored visually using light microscopy for morphological changes, and evaluated for neuronal cell markers (NeuN, PSD95 and NCAM) during the differentiation process [12, 14].

### Pharmaceuticals treatments

Stock solutions (10 mM) of fluoxetine (FLX; Sigma F133; Active metabolite), venlafaxine (VNX; Sigma D2069; ​Active metabolite) and carbamazepine (CBZ; Sigma C4206; ​Active metabolite), and 1 mM stock solution of valproic acid (VPA; Santa Cruz Biotechnology sc211393; Active metabolite) were prepared in dimethyl sulfoxide (DMSO). After differentiation for two weeks, cells were treated with a mixture composed of (MIX: FLX 10 μg/l; VNX 50 μg/l; CBZ 100 μg/l) [2], and Valproate (VPA: 4.9 mg/l) [15]. Control cells were treated with DMSO (vehicle) only, and the final concentration of DMSO in the cultures was 0.05 %. To examine if psychoactive pharmaceuticals in drinking water at environmental concentrations could induce neuronal gene expression, we chose to treat neuronal cells with the mixture of three pharmaceuticals (fluoxetine, carbamazepine and venlafaxine). Cells were treated in three replicates with the pharmaceuticals for 48 h in EMEM media without FBS to avoid any binding of pharmaceuticals with the serum proteins. All of the treatments were shown not to affect overall cell viability with respect to control (no treatment), based on the adherent nature of the monolayers and the result from CyQuant Viability Assay [16]. After treating cells for 48 h, they were collected with Versene solution (Gibco).

### RNA extraction, cDNA synthesis and sequencing

After 48 h of exposure, cells were collected with trypsin, centrifuged, and RNA was extracted using Qiagen RNeasy Plus Mini Kit (74134) according to the manufacturer’s protocol. Total RNA concentration was determined using NanoDrop (Thermo Scientific), and the RNA integrity value (RIN) was analyzed on Agilent 2100 Bioanalyzer (Agilent, Santa Clara, CA). RNA was quantified with Qubit spectrophotometer (Life technologies). Using Illumina Tru-seq stranded total RNA kit, cDNA library was prepared in following steps: RiboZero depletion and fragmentation, first and second strand cDNA synthesis, adenylation of 3′ ends, adapter ligation, PCR amplification, library validation using qPCR – Kapa Biosystems Library Quantification Kit, and normalization and pooling in preparation for cluster generation on MiSeq. Samples were then loaded onto flow cell (MiSeq Reagent kit v3 150 cycle) and sequenced on Illumina MiSeq 2 according to the manufacturer’s instructions.

### Bioinformatics data analysis

#### Quality control, alignment, and read counting

In total, three treatments (mixture, valproate, and control) with three replicates each were sequenced with five flow cells using Illumina MiSeq, which generated more than 10 M paired-end reads for each replicate (refer Additional file 1). The raw sequences in FASTQ files underwent quality control analysis using FastQC (http://www.bioinformatics.babraham.ac.uk/projects/fastqc). All the samples were sequenced at 75b read length and they all passed the quality check. We aligned the quality checked reads to human genome hg19 using TopHat version 2.0.11 [17]. Most of the reads (~90 %) had average base quality above Q30. The reference genome sequence, the bowtie index files for the reference genome sequence, and the gene annotation file were downloaded from Illumina iGenomes project (http://support.illumina.com/sequencing/sequencing_software/igenome.html). The number of reads that map to human known genes were counted by summerizeOverlaps function in GenomicAlignments R package [18]. Only the genes that have at least one read for all replicates were retained for downstream analysis.

#### Analysis of differential gene expression

Differentially expressed genes were identified using DESeq2 version 1.6.1 [19]. Differentially expressed genes from 2 groups (Mixture and Valproate) with respect to the control treatment were identified.

#### Gene set enrichment analysis

We used GAGE version 2.14.4 [20] for gene set enrichment analysis. We used GAGE to analyze gene sets that were analyzed previously in Fish brains [2, 6]. These gene sets are grouped as Neuronal development, regulation, growth; Neurological Disorders (ND); Autism spectrum disorders (ASD) [2, 6]. For extensive analysis, we also analyzed gene sets from MSigDB ‘C2’ and ‘C5’ (refer Additional file 2). Enrichment analyses were carried out using gage function using non-parametric Kolmogorov-Smirnov tests. We used GAGE package to identify significantly enriched (significantly up- and/or down-regulated; *P*-value < 0.01 and *Q*-value < 0.1) gene sets within mixture and valproate treatments.

## Results

### Patterns of psychoactive pharmaceuticals – induced in vitro gene expression in neuronal development, growth and regulation

We postulated that psychoactive pharmaceuticals at environmental concentrations would alter gene expression of neuronal systems. Support for this hypothesis would suggest that dysregulation of neuronal systems would result in altered neuronal circuits and may result in fewer neuronal connections. To address this question we used differentiated SK-N-SH neuroblastoma cells and treated them with the mixture (MIX: FLX-10 μg/l; VNX-50 μg/l; CBZ-100 μg/l), and valproate (VPA: 4.9 mg/l) in replicates of three samples for each treatment.

For the development collection, we observed six enriched gene sets (significantly down-regulated, *P*-value < 0.01, *Q*-value < 0.1) by MIX treatment. VPA treatment enriched the expression of seven gene sets (four gene sets down- and three gene sets up-regulated, Additional file 3). AXONOGENESIS, REGULATION OF NEUROGENESIS and SYNAPSE PART gene sets were enriched in both VPA and MIX treatments. And, SYNAPSE PART gene set was also enriched (up-regulated) in fish brains.

For the regulation collection, we observed 14 enriched gene sets (13 gene sets down- and one gene set up-regulated, *P*-value < 0.01, *Q*-value < 0.1) by MIX treatment. VPA treatment enriched the expression of eight gene sets (up-regulated, Additional file 3). All eight up-regulated gene sets in VPA treatment were down-regulated in MIX treatment. Among those sets, two gene sets (NEUROTRANSMITTER_BINDING and SYNAPSE) were also enriched in fish brains.

For the growth collection, we observed eight enriched gene sets (all down-regulated, *P*-value < 0.01, *Q*-value < 0.1) by MIX treatment. VPA treatment enriched the expression of five gene sets (up-regulated, Additional file 3). All five up-regulated gene sets in VPA treatment were down-regulated in MIX treatment. Two gene sets (AXON and NEURON_PROJECTION) were also enriched in fish brains.

### Patterns of psychoactive pharmaceuticals – induced in vitro gene expression in neurological disorders (ND) and ASD groups

We then sought to determine if altered in vitro gene expression was associated with neurological disorders and ASD. To accomplish this, we analyzed and compared the gene expression of already published ND and ASD gene sets in MIX and VPA treatments.

For the ND collection, we observed six enriched gene sets (four down- and two up-regulated, *P*-value < 0.01, *Q*-value < 0.1) by MIX treatment. VPA treatment enriched the expression of three gene sets (two gene sets down- and one gene set up-regulated, Additional file 3). AUTISM_IDIOPATHIC and PARKINSONS gene sets were enriched in both VPA and MIX treatments as well as in fish brains.

For the ASD collection, we observed two enriched gene sets (both up-regulated, *P*-value < 0.01, *Q*-value < 0.1) by MIX treatment. VPA treatment enriched the expression of five gene sets (down-regulated, Additional file 3). ASD_MILD gene set was enriched in both VPA and MIX treatments as well as in fish brains.

### Ranked gene lists from the mixture and valproate treatment

We sorted all genes from gene expression profiles in human neuronal cells treated with the MIX (FLX, VNX, CBZ) and valproate (VPA). In each treatment, we sequenced ~ 17,000 genes and analyzed their expression. We then ranked them based on their fold change expression within each treatment. Tables 1 and 2 show, the 50 most strongly up-and down-regulated genes. We also reported the fold-change score of genes which were presented on the fish microarray chip.

**Table 1:** Ranked gene list from the mixture treatment (50 most up- and down-regulated)

Up-regulated genes					Down-regulated genes				
Rank	Genes	Fold change	*P*-value	Fold change in fish brains	Rank	Genes	Fold change	*P*-value	Fold change in fish brains
1	*FAM129A*	3.25	0.000		17350	*ANGPTL4*	−3.36	0.000	
2	*CACNA2D3*	1.99	0.000	0.90	17349	*THBS1*	−2.19	0.000	0.34
3	*NUPR1*	1.97	0.002	−0.10	17348	*STARD13*	−2.17	0.000	−0.46
4	*GRM8*	1.90	0.000		17347	*DRP2*	−2.15	0.000	
5	*PTCHD1*	1.89	0.000		17346	*TNNT2*	−2.11	0.000	−0.23
6	*TMEM132C*	1.76	0.000		17345	*AQP10*	−2.10	0.000	
7	*DDIT3*	1.55	0.000	0.73	17344	*STRA6*	−1.97	0.000	−0.10
8	*XKR4*	1.53	0.010		17343	*PAPPA2*	−1.92	0.000	
9	*CHRNA10*	1.33	0.002		17342	*NEDD9*	−1.88	0.000	−1.21
10	*LPHN3*	1.24	0.004		17341	*GGTA1*	−1.85	0.001	
11	*SERPINF2*	1.23	0.008	0.15	17340	*SH3TC1*	−1.83	0.000	
12	*NKX3-1*	1.22	0.008		17339	*ADAMTSL2*	−1.80	0.000	−0.25
13	*HRK*	1.22	0.051		17338	*RGS13*	−1.78	0.000	
14	*SCARNA20*	1.22	0.009		17337	*ANO1*	−1.77	0.000	
15	*PABPC3*	1.21	0.006		17336	*LOC283731*	−1.75	0.000	
16	*KIT*	1.18	0.001	0.15	17335	*CMKLR1*	−1.73	0.000	0.86
17	*KIAA1045*	1.18	0.002	−0.12	17334	*VIP*	−1.71	0.000	
18	*DLL1*	1.15	0.013	0.05	17333	*HTR2B*	−1.69	0.000	
19	*C9orf150*	1.12	0.000		17332	*RAG2*	−1.67	0.004	0.20
20	*SNORA14A*	1.11	0.164		17331	*ACVRL1*	−1.67	0.000	
21	*B3GALT1*	1.09	0.134		17330	*VCAN*	−1.66	0.000	
22	*RIMBP3C*	1.09	0.096		17329	*C6*	−1.65	0.006	−1.11
23	*NOS1AP*	1.06	0.172	0.37	17328	*ENO3*	−1.64	0.000	−0.00
24	*GLYATL2*	1.06	0.021		17327	*TM4SF1*	−1.63	0.008	0.22
25	*STAC2*	1.04	0.259		17326	*PCTK3*	−1.62	0.004	
26	*CRYBB2*	1.04	0.020	0.01	17325	*FAM65C*	−1.60	0.000	
27	*SLC16A10*	1.01	0.167	−0.31	17324	*FLJ36208*	−1.60	0.000	
28	*CDH7*	1.01	0.031	−0.00	17323	*LOC400950*	−1.58	0.000	
29	*MYB*	1.01	0.030	−0.17	17322	*RARRES3*	−1.58	0.000	
30	*LCA5L*	1.00	0.176		17321	*SLCO4C1*	−1.53	0.000	
31	*ID4*	0.99	0.033	−0.17	17320	*PRAP1*	−1.52	0.022	
32	*MALAT1*	0.99	0.004		17319	*SLC18A1*	−1.51	0.000	
33	*SNAR-A3*	0.99	0.230		17318	*TFPI2*	−1.51	0.000	
34	*EDN1*	0.98	0.033		17317	*FBP1*	−1.50	0.000	0.43
35	*VSTM2A*	0.97	0.000		17316	*FGL1*	−1.49	0.001	
36	*HERV-FRD*	0.97	0.036		17315	*COL9A3*	−1.48	0.001	
37	*CNTD1*	0.97	0.037	−1.03	17314	*LRRTM1*	−1.48	0.015	0.25
38	*ELL2*	0.96	0.000	−0.30	17313	*BTK*	−1.48	0.000	−0.30
39	*SLC13A3*	0.96	0.027	0.02	17312	*SLC24A2*	−1.46	0.000	0.11
40	*DENND1B*	0.96	0.114	0.06	17311	*IRF6*	−1.45	0.000	0.17
41	*CYP2C18*	0.95	0.041		17310	*LOC392232*	−1.45	0.012	
42	*AMN*	0.95	0.041	−0.33	17309	*RD3*	−1.45	0.000	
43	*NCRNA00087*	0.95	0.301		17308	*DDC*	−1.43	0.000	
44	*FUT9*	0.94	0.042	−0.19	17307	*PRLHR*	−1.43	0.008	
45	*SCGB1D2*	0.92	0.047		17306	*NOTUM*	−1.42	0.052	−0.31
46	*TRIB3*	0.92	0.000	0.67	17305	*TMIGD2*	−1.41	0.008	
47	*MYBL1*	0.91	0.019		17304	*RTL1*	−1.40	0.048	−0.40
48	*ZNF726*	0.91	0.260		17303	*P2RX6*	−1.40	0.000	
49	*DLEU1*	0.90	0.173		17302	*DKK1*	−1.39	0.000	
50	*SLC7A11*	0.90	0.309	−0.41	17301	*TRIM9*	−1.38	0.000	

Table representing the list of 50 most up- and down-regulated genes in human neuronal cells treated with the mixture (FLX, VNX, CBZ). Genes were ranked based on their expression in fold-change in human cells. *P*-value represents the significance level of the expression change in the mixture treatment than control. Corresponding fold change of genes in fish brains is also reported in this table, where blank cells represents genes that are not found in the fish microarray chip

**Table 2:** Ranked gene list from the valproate treatment (50 most up- and down-regulated)

Up-regulated genes					Down-regulated genes				
Rank	Genes	Fold change	*P*-value	Fold change in fish brains	Rank	Genes	Fold change	*P*-value	Fold change in fish brains
1	*OPRK1*	4.03	0.000	−0.29	17673	*CMKLR1*	−2.81	0.000	0.86
2	*NEUROG2*	3.55	0.000		17672	*ADAMTS2*	−2.74	0.000	0.09
3	*VSNL1*	3.19	0.000	2.84	17671	*HIST1H4L*	−2.67	0.000	
4	*GALNAC4S-6ST*	3.09	0.000	0.96	17670	*ELFN1*	−2.44	0.000	
5	*POSTN*	2.93	0.000	1.22	17669	*COL4A2*	−2.39	0.000	−0.42
6	*EVX2*	2.86	0.000		17668	*GREM2*	−2.33	0.000	
7	*SERPINE1*	2.69	0.000	−1.45	17667	*FLJ45455*	−2.32	0.000	0.86
8	*ODZ1*	2.64	0.000		17666	*VCAN*	−2.31	0.000	
9	*DRD5*	2.34	0.000	−0.23	17665	*SOCS3*	−2.29	0.000	0.02
10	*CSGALNACT1*	2.33	0.001		17664	*TGFBI*	−2.27	0.000	−1.11
11	*HOXD13*	2.31	0.000		17663	*C11orf53*	−2.26	0.000	
12	*C3orf57*	2.29	0.000		17662	*SNAI1*	−2.17	0.000	−0.82
13	*B3GALT1*	2.29	0.000		17661	*LOC283480*	−2.11	0.000	
14	*SECTM1*	2.28	0.001		17660	*ID1*	−2.05	0.000	0.23
15	*PLAU*	2.20	0.001		17659	*PPP1R9A*	−2.03	0.000	−0.05
16	*PDE1A*	2.19	0.000		17658	*LOC646498*	−2.02	0.004	
17	*VIM*	2.14	0.000	0.25	17657	*RPS16P5*	−2.02	0.000	
18	*MME*	2.11	0.000		17656	*COL4A1*	−1.98	0.000	0.10
19	*KCNJ2*	2.09	0.000		17655	*GLT8D2*	−1.98	0.000	
20	*HS6ST2*	2.09	0.000	−0.15	17654	*CYP26A1*	−1.96	0.000	0.26
21	*SNORA42*	2.00	0.003		17653	*SLC10A1*	−1.95	0.004	
22	*RASEF*	1.99	0.000	0.31	17652	*PART1*	−1.94	0.000	
23	*APOL6*	1.93	0.000		17651	*OXTR*	−1.89	0.000	1.26
24	*NCRNA00164*	1.91	0.007		17650	*PTPLAD2*	−1.84	0.007	
25	*COLQ*	1.91	0.000		17649	*C9orf131*	−1.83	0.001	
26	*CACNA2D3*	1.90	0.000	0.90	17648	*FOS*	−1.80	0.000	−0.63
27	*DEGS2*	1.90	0.000		17647	*C9orf135*	−1.79	0.009	
28	*NHS*	1.89	0.000		17646	*GLP1R*	−1.79	0.000	
29	*PTER*	1.89	0.001	0.11	17645	*ETS1*	−1.79	0.000	−0.79
30	*EDIL3*	1.87	0.000	−0.41	17644	*NXPH3*	−1.79	0.000	
31	*INSM2*	1.85	0.000		17643	*LOC100128505*	−1.79	0.013	
32	*GFRA3*	1.82	0.000		17642	*KCTD12*	−1.76	0.000	0.49
33	*PROKR2*	1.82	0.000		17641	*ANGPTL4*	−1.74	0.000	
34	*GRM8*	1.81	0.001		17640	*FOSB*	−1.73	0.000	
35	*TLE4*	1.79	0.000		17639	*MPPED2*	−1.72	0.000	−0.62
36	*WNT5A*	1.77	0.001	−0.38	17638	*C11orf92*	−1.71	0.003	
37	*SMOC2*	1.77	0.000	−0.26	17637	*WDR38*	−1.69	0.001	
38	*VCAM1*	1.76	0.014	0.53	17636	*GALNT14*	−1.68	0.000	0.03
39	*FRMD4B*	1.74	0.000	0.10	17635	*LOC283143*	−1.67	0.002	
40	*PTRF*	1.74	0.000		17634	*ACCN1*	−1.66	0.002	
41	*DLK1*	1.70	0.000	0.69	17633	*C14orf53*	−1.66	0.002	
42	*PTCHD1*	1.67	0.001		17632	*TAPBPL*	−1.65	0.021	
43	*GPR126*	1.66	0.022		17631	*NRP1*	−1.63	0.000	1.12
44	*ANKDD1B*	1.65	0.000		17630	*BFSP2*	−1.63	0.002	
45	*MOXD1*	1.65	0.002	−1.00	17629	*SMAD7*	−1.63	0.000	0.08
46	*FAM111B*	1.65	0.000		17628	*HOPX*	−1.62	0.028	
47	*LOC728739*	1.63	0.002		17627	*ERBB4*	−1.62	0.000	
48	*CHRNA10*	1.62	0.002		17626	*PAPPA2*	−1.62	0.000	
49	*NELL1*	1.62	0.000		17625	*PCDHGB8P*	−1.62	0.011	
50	*NELL2*	1.62	0.000	−0.76	17624	*LOC284454*	−1.60	0.000	

Table representing the list of 50 most up- and down-regulated genes in human neuronal cells treated with the valproate. Genes were ranked based on their expression in fold- change in human cells. *P*-value represents the significance level of the expression change in the mixture treatment than control. Corresponding fold change of genes in fish brains is also reported in this table, where blank cells represents genes that are not found in the fish microarray chip

## Discussion

### Comparison between human MIX treatment and fish gene expression patterns

The results partially supported our first hypothesis that the MIX treatment on human SK-N-SH cell line induced gene sets enrichment patterns similar to that of the fish experiment following mixture treatment, although the degree of similarity was not high.

Among the neural circuit development gene sets, all the six significantly enriched gene sets in human neuronal cells following MIX treatment were enriched in a down-regulated manner; whereas in the fish experiment, the two significantly enriched sets were both up-regulated. We do not know why the direction was opposite, but we do notice that, one gene set, SYNAPSE PART, was enriched in both treatments [2]. Previous studies have found that altered expression of *NCAM*, *IRX3* and *NKX6.1* genes in SYNAPSE PART change the fate and position of neurons generated in the chick neural tube [21, 22]. Also within those down-regulated gene sets contains gene *PSD95* (*DLG4*) and *GABA*, which have recently been found to be associated with neurological disorders like autism by altering synaptic assembly [14, 23, 24].

In the growth group, we observed similar patterns where all enriched sets in MIX treatment were down-regulated and all those in the fish experiment were up-regulated. Among those sets, two were enriched in both treatments, AXON and NEURON PROJECTION. Other studies have found that genes within these two gene sets modulate the fate, lineage, and timing of neuronal development by playing a critical role in the formation and maturation of neural circuits [23, 25, 26].

In the regulation group, we observed that NEUROTRANSMITTER BINDING gene set was down-regulated in fish brains as well as in human cells [2]. This could be possible due to the therapeutic effect of fluoxetine (SSRI) in the mixture treatment [2]. Fluoxetine is known to reduce the re-uptake of serotonin by inhibiting monoamine transporters on the pre-synaptic neuronal membrane [27, 28]. Due to the longer availability of neurotransmitters in the synaptic cleft, the expression of serotonin receptors is down-regulated, thus a decrease in neurotransmitter binding [29]. Another gene set SYNAPSE was significantly up-regulated in fish brains [2], but down-regulated in treated human cells. This gene set was responsible for modulating wiring of neuronal circuits by controlling the number of synapse as well as organization of synaptic assembly and specificity [26, 30]. Altered synaptogenesis has been strongly considered as a potential mechanism in ASD pathogenesis [31, 32].

### Comparison between human MIX and VPA gene expression patterns

We used valproate (an anticonvulsant) to treat human neuronal cells as a positive control because prenatal exposure of valproate has been found to be strongly associated with autism [3] and valproate is known to induce autism-like phenotypes in mice [11]. Similarly, carbamazepine (presented in the mixture treatment) is a mood stabilizer and anticonvulsant [11, 33] and it also inhibits the epileptic effects in the brain by blocking sodium channels [11, 33]. By and large, the results support our second hypothesis that the MIX and VPA treatments change the RNA expression profile in similar ways, albeit in an interesting fashion, since the two treatments often enrich the same gene sets but in different directions.

In the development group of VPA treatment, we found four gene sets significantly down-regulated and three gene sets significantly up-regulated in human neuronal cells. Three gene sets (AXONOGENESIS, REGULATION OF NEUROGENESIS and SYNAPSE PART) were enriched in both mixture and valproate treatments, but in the opposite direction. Similar to fish gene expression, VPA treatment up-regulated SYNAPSE PART gene set [2]. This states that VPA exposure might be associated with disturbed neuronal fate and position [21, 22] as well as synaptic assembly [1, 22, 34].

In the growth group, we observed that five gene sets were up-regulated in the VPA treatment. Interestingly, the same five sets were all down-regulated in the MIX treatment. This suggested that both treatments disturbed the human gene expression in similar pathways despite the different directions. One gene set NEURON PROJECTION was up-regulated similar to fish brains [2]. In the regulation group, we noticed similar results. Eight gene sets were up-regulated in valproate treatment, but down-regulated in the mixture treatment. From this repeating phenomenon of opposite direction enrichment of the same gene set, we postulate a general pattern that the three pharmaceuticals (fluoxetine, venlafaxine and carbamazepine) and valproate dysregulate the same pathways in different directions. However, more investigation is needed to confirm or disprove this postulation. We also plotted multi-dimensional scaling using edgeR [35] and observed that mixture treatment samples were somewhat closer to valproate samples than control samples (refer Additional file 1). However, MIX and VPA samples were still apart, confirming that these two treatments did not exhibit similar response.

### Association of human MIX and VPA gene expression patterns with neurological disorders

To determine the extent to which altered gene expression in both MIX and VPA treatments were associated with neurological disorders (including ASD), we examined the expression of the already-published gene sets from neurological disorders (ND) and ASD groups in both treatments. We also compared these to their corresponding expression pattern in fish brains. For ND group, MIX and VPA treatments altered PARKINSONS and Autism_Idiopathic gene sets significantly but in different directions. Interestingly, mixture treatment of human cells and fish brains up-regulated Autism_Idiopathic gene set [6]. For another ASD group, MIX treatment up-regulated two gene sets (ASD_2Class and ASD_Mild) similar to fish brains [6]. On the other side, VPA treatment down-regulated three gene sets (ASD_2Class, ASD_Mild, ASD_Savant) in a different direction compared to the expression in fish brains [6]. These expression patterns stated that VPA and MIX treatments of human cells exhibited a similar response to neurological disorders (including ASD), suggesting a common induction effect.

### Insights into important genes in both MIX and VPA treatments

We sought to identify important, or novel genes that were significantly up- and down-regulated in human neuronal cells treated with MIX and VPA. We generated ranked lists of genes based on their fold change, and tabulated the most 50 strongly up- and down-regulated genes (Table 1 and 2). We also compared genes from ranked lists with the ones from fish microarray data. For MIX treatment, we noticed four genes (*NUPR1*, *RTL1*, *THBS1* and *HTR2B*) which could be considered important and novel. The thrombospondin (*THBS1*) gene plays an important role in synaptogenesis in the developing brain [36]. Recent association studies have found that both rare and common variants of this gene are associated with autism [36]. Although this gene was found to be down-regulated by two-fold, it was up-regulated in fish brains under similar mixture treatment [2]. Another important gene, *HTR2B*, which codes for serotonin receptor 2B, was down-regulated by ~ two-fold in human cells. Similar serotonin receptor genes were also found to be down-regulated in fish brains. Moreover, recent protein studies in our lab showed that HTR2B protein was down-regulated in the same mixture (FLX, VNX, CBZ) treatment. This mechanism is explained by the drug effect of fluoxetine (SSRI), which provides more neurotransmitter in the synaptic cleft, thus reducing serotonin receptors [29].

In the ranked list of genes by valproate (VPA) treatment, we found three genes (*VSNL1*, *PTER* and *OXTR*) of particular importance. *VSNL1* gene, which encodes for visinin-like protein 1 in humans, modulates neuronal morphology by controlling the key signaling pathways in CNS. This gene was up-regulated by three-fold in the valproate treatment. We observed similar up-regulation of this gene in fish brains following mixture (FLX, VNX, CBZ) treatment. Other studies have recently found the association of single-nucleotide polymorphisms (SNPs) in the *VSNL1* gene with neurological disorders like schizophrenia [37]. Another important gene, oxytocin receptor (*OXTR*) was found to be down-regulated by two-fold in human cells treated with valproate (Table 2). However, this gene was up-regulated in fish brains exposed to the mixture of psychoactive pharmaceuticals [2]. Moreover, recent protein studies in our lab showed an increased expression of OXTR in the same cells treated with carbamazepine and the mixture (FLX, VNX, CBZ). Other studies have shown that OXTR serves as an anxiolytic agent by modulating serotonin release in serotonergic neurons of the raphe nuclei [38].

## Conclusions

To investigate the environmental trigger for idiopathic autism, we focused on psychoactive pharmaceuticals, potential environmental contaminants that have been detected in drinking water. We found that psychoactive pharmaceuticals altered the gene expression of neuronal systems in vitro at environmental concentrations. These altered gene expressions are associated with potential neurological disorders by playing a key role in the formation, growth and regulation of neurons. Our data suggests that psychoactive pharmaceuticals might disrupt neuronal connections by altering the gene expression associated with neuronal growth, development and regulation.

## Additional files

Additional file 1: R-programming code for RNA-Seq analysis and multi-dimensional scaling (MDS) function. The file contains R-code for analysis of RNA-Seq data for mixture and valproate treatments. This file also includes the code for plotMDS function for using multi-dimensional scaling. (DOCX 61 kb) Additional file 2: Complete data of gene sets analyzed. Excel file containing a complete data of all gene sets (neuronal development, growth, regulation; neurological disorders and ASD group) that were enriched (up- and down-regulated) in mixture (carbamazepine, fluoxetine and venlafaxine) and valproate treatments. We also reported *P*- values and *Q*-values for individual gene sets. (XLSX 1621 kb) Additional file 3: Descriptions of gene sets and results of analyses. This file contains table that represents the analyses of gene sets within neuronal systems (a. Development, b. Regulation, c. Growth), (d) Neurological Disorders, and (e) ASD groups. Source indicates the database from where the gene set was derived, Gene Ontology (GO) or Molecular Signatures Database (MSigDB). Size represents the number of genes in each gene set. MIX_UP, MIX_DOWN, VPA_UP and VPA_DOWN indicates gene sets which were found up-regulated in the mixture, down-regulated in the mixture, up-regulated in the valproate, and down-regulated in the valproate treatments, respectively. Gene sets with *P*-value < 0.01 and *Q*-value < 0.1 were considered as statistically significantly enriched. For non-significant gene sets, refer Additional file 2 for *P*-values and *Q*-values. Enriched gene sets in human cells and corresponding scores are marked in bolds. Enriched gene sets in fish brains are italicized and marked in parentheses. (DOCX 124 kb)
