# Supplementary material for: Psychoactive pharmaceuticals at environmental concentrations induce in vitro gene expression associated with neurological disorders
Source: BMC Genomics. 2016 Jun 29;17(Suppl 3):435. doi: 10.1186/s12864-016-2784-1 (PMC4943479; doi:10.1186/s12864-016-2784-1)
Supplement: Additional file 1: — R-programming code for RNA-Seq analysis and multi-dimensional scaling (MDS) function. The file contains R-code for analysis of RNA-Seq data for mixture and valproate treatments. This file also includes the code for plotMDS function for using multi-dimensional scaling. (DOCX 61 kb) [file 12864_2016_2784_MOESM1_ESM.docx]

- **Supplementary Table S1**

Number of paired-end reads generated by Illumina MiSeq for each sample

| Sample | Biological replicate | Number of paired-end reads |
| --- | --- | --- |
| Control | 1 | 13,879,138 |
|  | 2 | 14,115,486 |
|  | 3 | 14,747,099 |
| Mixture | 1 | 11,666,138 |
|  | 2 | 10,035,614 |
|  | 3 | 15,817,684 |
| Valproate | 1 | 18,336,265 |
|  | 2 | 19,043,551 |
|  | 3 | 17,566,449 |

- **R programming code of the analysis**

**Mixture treatment:**

# Read the table that contains the number of reads for each gene.

cnts=read.table("mixture.counts.txt", header=T, row.names=1)

# Normalize the number of reads

libsizes=colSums(cnts)

size.factor=libsizes/exp(mean(log(libsizes)))

cnts.norm=t(t(cnts)/size.factor)

cnts.norm=log2(cnts.norm+8)

# the first 3 samples are control, and the last 3 samples are treatment.

ref.idx=1:3

samp.idx=4:6

# read the gene set file ND.gmt

library(gage)

data(egSymb)

geneset.file="ND.correct.gmt"

gene.set = readList(geneset.file)

# Convert the gene symbols to Entrez gene ID for the get set

gene.set = lapply(gene.set, sym2eg)

# Run gage for gene set enrichment analysis

ND.p = gage(cnts.norm, gsets=gene.set, ref=ref.idx, samp=samp.idx, saaTest=gs.KSTest)

ND.sig = sigGeneSet(ND.p, outname="mixture.ND")

# read the gene set file development.gmt

geneset.file = "development.correct.gmt"

gene.set=readList(geneset.file)

gene.set = lapply(gene.set, sym2eg)

development.p = gage(cnts.norm, gsets=gene.set, ref=ref.idx, samp=samp.idx, saaTest=gs.KSTest)

development.sig = sigGeneSet(development.p, outname="mixture.development")

# read the gene set file growth.gmt

geneset.file = "growth.correct.gmt"

gene.set=readList(geneset.file)

geneset=lapply(gene.set, sym2eg)

growth.p = gage(cnts.norm, gsets=gene.set, ref=ref.idx, samp=sampl.idx, saaTest=gs.KSTest)

growth.sig = sigGeneSet(growth.p, outname="mixture.growth")

# read the gene set file regulation.gmt

geneset.file = "regulation.correct.gmt"

gene.set=readList(geneset.file)

gene.set=lapply(gene.set, sym2eg)

regulation.p = gage(cnts.norm, gsets=gene.set, ref=ref.idx, samp=samp.idx, saaTest=gs.KSTest)

regulation.sig = sigGeneSet(regulation.p, outname="mixture.regulation")

**Valproate treatment:**

# Read the table that contains the number of reads for each gene.

cnts=read.table("vpa.counts.txt", header=T, row.names=1)

# Normalize the number of reads

libsizes=colSums(cnts)

size.factor=libsizes/exp(mean(log(libsizes)))

cnts.norm=t(t(cnts)/size.factor)

cnts.norm=log2(cnts.norm+8)

# the first 3 samples are control, and the last 3 samples are treatment.

ref.idx=1:3

samp.idx=4:6

# read the gene set file ND.gmt

library(gage)

data(egSymb)

geneset.file="ND.correct.gmt"

gene.set = readList(geneset.file)

# Convert the gene symbols to Entrez gene ID for the get set

gene.set = lapply(gene.set, sym2eg)

# Run gage for gene set enrichment analysis

ND.p = gage(cnts.norm, gsets=gene.set, ref=ref.idx, samp=samp.idx, saaTest=gs.KSTest)

ND.sig = sigGeneSet(ND.p, outname="vpa.ND")

# read the gene set file development.gmt

geneset.file = "development.correct.gmt"

gene.set=readList(geneset.file)

gene.set = lapply(gene.set, sym2eg)

development.p = gage(cnts.norm, gsets=gene.set, ref=ref.idx, samp=samp.idx, saaTest=gs.KSTest)

development.sig = sigGeneSet(development.p, outname="vpa.development")

# read the gene set file growth.gmt

geneset.file = "growth.correct.gmt"

gene.set=readList(geneset.file)

geneset=lapply(gene.set, sym2eg)

growth.p = gage(cnts.norm, gsets=gene.set, ref=ref.idx, samp=sampl.idx, saaTest=gs.KSTest)

growth.sig = sigGeneSet(growth.p, outname="vpa.growth")

# read the gene set file regulation.gmt

geneset.file = "regulation.correct.gmt"

gene.set=readList(geneset.file)

gene.set=lapply(gene.set, sym2eg)

regulation.p = gage(cnts.norm, gsets=gene.set, ref=ref.idx, samp=samp.idx, saaTest=gs.KSTest)

regulation.sig = sigGeneSet(regulation.p, outname="vpa.regulation")

- **Multi-Dimensional Scaling (MDS)**

MDS plot: The plot represents the spatial location of samples from three treatments, which are mixture, valproate and control. This plot was generated by edgeR function in R programming.
