# Supplementary material for: Psychoactive pharmaceuticals at environmental concentrations induce in vitro gene expression associated with neurological disorders
Source: BMC Genomics. 2016 Jun 29;17(Suppl 3):435. doi: 10.1186/s12864-016-2784-1 (PMC4943479; doi:10.1186/s12864-016-2784-1)
Supplement: Additional file 3: — Descriptions of gene sets and results of analyses. This file contains table that represents the analyses of gene sets within neuronal systems (a. Development, b. Regulation, c. Growth), (d) Neurological Disorders, and (e) ASD groups. Source indicates the database from where the gene set was derived, Gene Ontology (GO) or Molecular Signatures Database (MSigDB). Size represents the number of genes in each gene set. MIX_UP, MIX_DOWN, VPA_UP and VPA_DOWN indicates gene sets which were found up-regulated in the mixture, down-regulated in the mixture, up-regulated in the valproate, and down-regulated in the valproate treatments, respectively. Gene sets with P-value < 0.01 and Q-value < 0.1 were considered as statistically significantly enriched. For non-significant gene sets, refer Additional file 2 for P-values and Q-values. Enriched gene sets in human cells and corresponding scores are marked in bolds. Enriched gene sets in fish brains are italicized and marked in parentheses. (DOCX 124 kb) [file 12864_2016_2784_MOESM3_ESM.docx]

## Descriptions of gene sets and results of analyses

| Source | Gene set name | Size | Function/Description | MIX_UP | MIX_DOWN | VPA_UP | VPA_DOWN |
| --- | --- | --- | --- | --- | --- | --- | --- |
| a. *Development collection, containing 20 sets* | | | | | | | |
| GO:0007409 | **AXONOGENESIS** | 378 | Formation of long process of a neuron, carrying outgoing action potential |  | **p< 0.01**  **q< 0.1** | **p< 0.01**  **q< 0.1** | **p< 0.01**  **q< 0.1** |
| GO:0007420 | **BRAIN_DEVELOPMENT** | 255 | Maturation of the brain structure |  |  |  | **p< 0.01**  **q< 0.1** |
| GO:0021954 | CENTRAL NERVOUS SYSTEM NEURON DEVELOPMENT | 29 | Progression and functional differentiaion of a neuron whose cell body lies in CNS |  |  |  |  |
| GO:0007417 | **CENTRAL_NERVOUS_SYSTEM_DEVELOPMENT** | 378 | Progression and maturation of CNS |  |  |  | **p< 0.01**  **q< 0.1** |
| GO:0021545 | CRANIAL NERVE DEVELOPMENT | 20 | Progression and formation of cranial nerves |  |  |  |  |
| GO:0016358 | **DENDRITE_DEVELOPMENT** | 58 | Development of dendrites |  | **p< 0.01**  **q< 0.1** |  |  |
| GO:0007272 | *(ENSHEATHMENT OF NEURONS)* | 43 | Formation of the insulated part of a neuron |  |  |  |  |
| GO:0030900 | FOREBRAIN_DEVELOPMENT | 142 | Progression and formation of forebrain |  |  |  |  |
| GO:0048699 | **GENERATION_OF_NEURONS** | 72 | Production of neuroblasts and differentiation into neurons |  |  |  | **p< 0.01**  **q< 0.1** |
| GO:0030902 | HIND BRAIN DEVELOPMENT | 43 | Progression and formation of hindbrain |  |  |  |  |
| GO:0050768 | NEGATIVE REGULATION OF NEUROGENESIS | 47 | Reduces the rate of neurogenesis |  |  |  |  |
| GO:0007399 | **NERVOUS_SYSTEM_DEVELOPMENT** | 328 | Progression and formation of the nervous tissue |  | **p< 0.01**  **q< 0.1** |  |  |
| GO:0021915 | *(NEURAL_TUBE_DEVELOPMENT)* | 68 | Progression and formation of the neural tube |  |  |  |  |
| GO:0022008 | NEUROGENESIS | 17 | Generation of new cells |  |  |  |  |
| GO:0048666 | NEURON_DEVELOPMENT | 25 | Progression and formation of the neuron |  |  |  |  |
| GO:0050769 | **POSITIVE REGULATION OF NEUROGENESIS** | 67 | Increases the rate of neurogenesis |  | **p< 0.01**  **q< 0.1** |  |  |
| GO:0050772 | POSITIVE_REGULATION_OF_AXONOGENESIS | 31 | Increases the rate of axonogenesis |  |  |  |  |
| GO:0050767 | **REGULATION_OF_NEUROGENESIS** | 193 | Controls the rate of neurogenesis |  | **p< 0.01**  **q< 0.1** | **p< 0.01**  **q< 0.1** |  |
| GO:0050808 | *(SYNAPSE_ORGANISATION)* | 62 | Organizes the synaptic assembly or arrangement |  |  |  |  |
| GO:0044456 | ***(SYNAPSE_PART)*** | 228 | Formation of a junction between a neuron and another neuron, or fiber, or glial cell |  | **p< 0.01**  **q< 0.1** | **p< 0.01**  **q< 0.1** |  |
| b. *Regulation collection, containing 22 sets* | | | | | | | |
| GO:0043679 | **AXON_TERMINUS** | 33 | Formation of the neuron terminal part that controls neurotransmitter secreation |  | **p< 0.01**  **q< 0.1** | **p< 0.01**  **q< 0.1** |  |
| GO:0008066 | **GLUTAMATE RECEPTOR ACTIVITY** | 25 | Binding of glutamate to the neuron |  | **p< 0.01**  **q< 0.1** |  |  |
| MSigDB C2 CPG | **HSA04080_NEUROACTIVE_LIGAND_RECEPTOR_INTERACTION** | 45 | Interaction of neuro-active ligand-receptor |  | **p< 0.01**  **q< 0.1** | **p< 0.01**  **q< 0.1** |  |
| MSigDB C2 CPG | LU_AGING_BRAIN_DN | 232 | Genes found down regulated in the frontal cortex of old subjects |  |  |  |  |
| MSigDB C2 CPG | *(LU_AGING_BRAIN_UP)* | 132 | Genes found up regulated in the frontal cortex of old subjects |  |  |  |  |
| GO:0007270 | NERVE-NERVE SYNAPTIC TRANSMISSION | 44 | Communicates between neurons |  |  |  |  |
| GO:0006836 | **NEUROTRANSMITTER TRANSPORT** | 97 | Controls the movement of neurotransmitters |  | **p< 0.01**  **q< 0.1** |  |  |
| GO:0042165 | ***(NEUROTRANSMITTER_BINDING)*** | 47 | Controls the impulse from a neuron to other cells |  | **p< 0.01**  **q< 0.1** | **p< 0.01**  **q< 0.1** |  |
| GO:0042133 | **NEUROTRANSMITTER_METABOLIC_PROCESS** | 19 | Controls chemical pathways and reactions of neurotransmitters |  | **p< 0.01**  **q< 0.1** |  |  |
| GO:0030594 | **NEUROTRANSMITTER_RECEPTOR_ACTIVITY** | 40 | Controls the binding of neurotransmitter to the cell |  | **p< 0.01**  **q< 0.1** |  |  |
| GO:0007269 | *(NEUROTRANSMITTER_SECRETION)* | 68 | Controls the secretion of neurotransmitters |  |  |  |  |
| GO:0050433 | REGULATION OF CATECHOLAMINE SECRETION | 17 | Controls the secreation of catecholamines |  |  |  |  |
| GO:0048168 | REGULATION OF NEURONAL SYNAPTIC PLASTICITY | 27 | Controls the neuronal plasticity |  |  |  |  |
| GO:0051969 | **REGULATION OF TRANSMISSION OF NERVE IMPULSE** | 105 | Controls the transmission of a neuron impulse |  | **p< 0.01**  **q< 0.1** | **p< 0.01**  **q< 0.1** |  |
| GO:0019228 | *(REGULATION_OF ACTIONPOTENTIAL_IN_NEURON)* | 53 | Controls the action potential of a neuron |  |  |  |  |
| GO:0031644 | **REGULATION_OF_NEUROLOGICAL_SYSTEM_PROCESS** | 111 | Controls the fate of a neurological process |  | **p< 0.01**  **q< 0.1** | **p< 0.01**  **q< 0.1** |  |
| GO:0001505 | **REGULATION_OF_NEUROTRANSMITTER_LEVELS** | 90 | Controls the level of neurotransmitters |  | **p< 0.01**  **q< 0.1** |  |  |
| GO:0005484 | **SNAP RECEPTOR ACTIVITY** | 19 | Modulates the fusion of membranes | **p< 0.01**  **q< 0.1** |  |  |  |
| GO:0000149 | *(SNARE BINDING)* | 26 | Helps in the interaction of SNARE protein |  |  |  |  |
| GO:0045202 | ***(SYNAPSE)*** | 319 | Formation of the junction of one neuron to others |  | **p< 0.01**  **q< 0.1** | **p< 0.01**  **q< 0.1** |  |
| GO:0007268 | **SYNAPTIC_TRANSMISSION** | 288 | Mediates the transmission between synapses |  | **p< 0.01**  **q< 0.1** | **p< 0.01**  **q< 0.1** |  |
| GO:0019226 | **TRANSMISSION_OF_NERVE_IMPULSE** | 336 | Mediates the depolarization and repolarization of a nerve impulse |  | **p< 0.01**  **q< 0.1** | **p< 0.01**  **q< 0.1** |  |
| c. *Growth collection, containing 16 sets* | | | | | | | |
| MSigDB C2 CPG | *(APPEL_IMATINIB_RESPONSE)* | 28 | Up-regulation by imatinib during dendritic cell differentiation |  |  |  |  |
| GO:0030424 | ***(AXON)*** | 158 | Mediates the formation of long process of a neuron |  | **p< 0.01**  **q< 0.1** |  |  |
| GO:0008366 | AXON_ENSHEATHMENT | 43 | Formation of the insulated part of a neuron |  |  |  |  |
| GO:0048675 | AXON_EXTENSION | 42 | Mediates the growth of the long process of a neuron |  |  |  |  |
| GO:0007411 | **AXON_GUIDANCE** | 282 | Mediates the migration of the long process of a neuron |  | **p< 0.01**  **q< 0.1** |  |  |
| GO:0033267 | **AXON_PART** | 63 | Mediates the cell projection of a neuron |  | **p< 0.01**  **q< 0.1** | **p< 0.01**  **q< 0.1** |  |
| GO:0048667 | **CELL MORPHOGENESIS INVOLVED IN NEURON DIFFERENTIATION** | 403 | Mediates the formation of neuron structures |  | **p< 0.01**  **q< 0.1** | **p< 0.01**  **q< 0.1** |  |
| GO:0021953 | CENTRAL NERVOUS SYSTEM NEURON DIFFERENTIATION | 54 | Modulates the formation of differentiation of CNS |  |  |  |  |
| GO:0042551 | NEURON MATURATION | 18 | Meidates the maturation of a neuron |  |  |  |  |
| GO:0048812 | **NEURON PROJECTION MORPHOGENESIS** | 408 | Controls the formation and organization of neuron projection |  | **p< 0.01**  **q< 0.1** | **p< 0.01**  **q< 0.1** |  |
| GO:0070997 | NEURON_DEATH | 93 | Mediates the neuron death |  |  |  |  |
| GO:0030182 | **NEURON_DIFFERENTIATION** | 61 | Mediates the differentiation of a neuron |  | **p< 0.01**  **q< 0.1** |  |  |
| GO:0043005 | ***(NEURON_PROJECTION)*** | 322 | Controls the projection of a neuron |  | **p< 0.01**  **q< 0.1** | **p< 0.01**  **q< 0.1** |  |
| GO:0043523 | *(REGULATION OF NEURON APOPTOSIS)* | 76 | Mediates the occurrence/rate of neuron death by apoptosis |  |  |  |  |
| GO:0045664 | **REGULATION OF NEURON DIFFERENTIATION** | 156 | Mediates the occurrence/rate of neuron differentiation |  | **p< 0.01**  **q< 0.1** | **p< 0.01**  **q< 0.1** |  |
| GO:0007416 | SYNAPSE ASSEMBLY | 39 | Organizes the assembly of a synapse |  |  |  |  |
| d. *Neurological Disorders (ND) collection, containing 12 sets* | | | | | | | |
| GSE12457 | **ADHD_down** | 32 | Down-regulated genes by molecular alterations in genetic and environmental rat models of ADHD |  | **p< 0.01**  **q< 0.1** |  |  |
| GSE12457 | **ADHD_up** | 44 | Up-regulated genes by molecular alterations in genetic and environmental rat models of ADHD |  | **p< 0.01**  **q< 0.1** |  |  |
| GSE1297 | **ALZHEIMERS** | 308 | Genes upregulated in the CA1 region of the hippocampus in Alzheimer’s disease | **p< 0.01**  **q< 0.1** |  |  |  |
| GSE7329 | ASD_Secondary | 49 | Genes expressed in lymphoblastoid cells from individuals with fragile X syndrome |  |  |  |  |
|  | ***(Autism_idiopathic)*** | 439 | Combination of Chakrabarti, Hu and ASD_2class; excluding duplicates | **p< 0.01**  **q< 0.1** |  |  | **p< 0.01**  **q< 0.1** |
|  | Bipolar | 57 | Upregulated genes in individuals with bipolar disorder |  |  |  |  |
| GSE12654 | Depression | 38 | Upregulated genes in individuals with depression |  |  |  |  |
|  | MS_Bomprezzi | 34 | Genes expressed in peripheral blood mononuclear cells from individuals with MS |  |  |  |  |
| GSE17393 | ***(MS_gilli)*** | 320 | Altered genes from non-pregnant MS patients |  |  |  | **p< 0.01**  **q< 0.1** |
|  | ***(PARKINSONS)*** | 130 | Genes associated with Parkinsons |  | **p< 0.01**  **q< 0.1** | **p< 0.01**  **q< 0.1** |  |
|  | RETT | 26 | Upregulated genes in females with Rett syndrome |  |  |  |  |
|  | **Schizophrenia** | 29 | Differentially expressed proteins in the brains of SCZ patients |  | **p< 0.01**  **q< 0.1** |  |  |
| e. *ASD collection, containing 8 sets* | | | | | | | |
| GSE15402 | ***(ASD_2Class)*** | 354 | Differentially expressed genes (significantly) from a SAM 2-class analysis of the data from combined autistic samples and neurotypical controls | **p< 0.01**  **q< 0.1** |  |  | **p< 0.01**  **q< 0.1** |
| GSE15402 | ***(ASD_Mild)*** | 306 | Differentially expressed genes (significantly) from a SAM 2-class analysis of the data from mild autistic samples and neurotypical controls | **p< 0.01**  **q< 0.1** |  |  | **p< 0.01**  **q< 0.1** |
|  | ***(ASD_Savant)*** | 71 | Differentially expressed genes (significantly) from a SAM 2-class analysis |  |  |  | **p< 0.01**  **q< 0.1** |
| GSE15402 | ASD_Severe | 176 | Differentially expressed genes (significantly) from a SAM 2-class analysis of the data from the group of individuals with severe language impairment and neurotypical controls |  |  |  |  |
| GSE15402 | *(ASD_Shared)* | 67 | Common genes from GSE15402 sets |  |  |  |  |
|  | **Chakrabarti** | 54 | Genes associated to neural growth, sex steroids and social impairment behavior related to autistic traits and Asperger’s sysndrome; excluding severe language impairment |  |  |  | **p< 0.01**  **q< 0.1** |
|  | Hu | 30 | Genes examined by ASD individuals with severe language impairment vs. neurotypical individuals |  |  |  |  |
|  | **Pinto** | 81 | Genes associated with ASD genetic susceptibility |  |  |  | **p< 0.01**  **q< 0.1** |
